# Silymarin Induces Insulin Resistance through an Increase of Phosphatase and Tensin Homolog in Wistar Rats

**DOI:** 10.1371/journal.pone.0084550

**Published:** 2014-01-03

**Authors:** Kai-Chun Cheng, Akihiro Asakawa, Ying-Xiao Li, Hsien-Hui Chung, Haruka Amitani, Takatoshi Ueki, Juei-Tang Cheng, Akio Inui

**Affiliations:** 1 Department of Psychosomatic Internal Medicine, Kagoshima University Graduate School of Medical and Dental Sciences, Kagoshima, Japan; 2 Institute of Basic Medical Sciences, College of Medicine, National Cheng Kung University, Tainan City, Taiwan; 3 Department of Neuroanatomy, Hamamatsu University School of Medicine. Hamamatsu, Japan; 4 Department of Medical Research, Chi-Mei Medical Center, Yong Kang, Tainan City, Taiwan; State University of Rio de Janeiro, Biomedical Center, Institute of Biology, Brazil

## Abstract

**Background and aims:**

Phosphatase and tensin homolog (PTEN) is a phosphoinositide phosphatase that regulates crucial cellular functions, including insulin signaling, lipid and glucose metabolism, as well as survival and apoptosis. Silymarin is the active ingredient in milk thistle and exerts numerous effects through the activation of PTEN. However, the effect of silymarin on the development of insulin resistance remains unknown.

**Methods:**

Wistar rats fed fructose-rich chow or normal chow were administered oral silymarin to identify the development of insulin resistance using the homeostasis model assessment of insulin resistance and hyperinsulinemic- euglycemic clamping. Changes in PTEN expression in skeletal muscle and liver were compared using western blotting analysis. Further investigation was performed in L6 cells to check the expression of PTEN and insulin-related signals. PTEN deletion in L6 cells was achieved by small interfering ribonucleic acid transfection.

**Results:**

Oral administration of silymarin at a dose of 200 mg/kg once daily induced insulin resistance in normal rats and enhanced insulin resistance in fructose-rich chow-fed rats. An increase of PTEN expression was observed in the skeletal muscle and liver of rats with insulin resistance. A decrease in the phosphorylation of Akt in L6 myotube cells, which was maintained in a high-glucose condition, was also observed. Treatment with silymarin aggravated high-glucose-induced insulin resistance. Deletion of PTEN in L6 cells reversed silymarin-induced impaired insulin signaling and glucose uptake.

**Conclusions:**

Silymarin has the ability to disrupt insulin signaling through increased PTEN expression. Therefore, silymarin should be used carefully in type-2 diabetic patients.

## Introduction

Hepatic pathologies, ranging from hepatic steatosis to steatohepatitis, fibrosis, and cirrhosis are commonly associated with metabolic disorders such as insulin resistance and dyslipidemia [1]. Obesity and metabolic syndrome are major etiological factors that contribute to the development of severe liver diseases [2], [3]. Although the development of metabolic and hepatic diseases is highly correlated, the effects of the medications prescribed for hepatic protection on glucose homeostasis remain unknown.

Milk thistle (*Silybum marianum*) is a dietary supplement that provides hepatic protection against drug- or alcohol-related injury [4]. Silymarin is an active mixture of flavonolignane diastereomers found in milk thistle extract, and reportedly acts as a strong antioxidant and free radical scavenger [5], [6], as well as exerts a liver-protective action without notable adverse effects. In addition, silymarin has demonstrated an inhibitory effect on multiple cancer cell lines, including prostate, lung, colon, skin, and bladder cancers [7]–[12], as well as hepatocellular carcinoma [13], [14]. The mechanism for the silymarin-mediated anti-tumorigenic effect is associated with increased activity of phosphatase and tensin homolog (PTEN) and the decreased phosphorylation of Akt [13].

The PTEN protein is a phosphoinositide phosphatase that dephosphorylates the phosphatidylinositol 3,4-bisphosphate and phosphatidylinositol 3,4,5-trisphosphate second messengers on the 3′-position of the inositol ring [15]. PTEN is frequently mutated in hepatocellular carcinoma [16]. PTEN antagonizes phosphoinositide 3-kinase (PI3K) activation and acts as a potent regulator of growth factor signaling in the insulin signal pathway [17]. PTEN-specific deletion in muscle improves skeletal muscle insulin sensitivity and to protect mice from insulin resistance [18]. Treatment with PTEN antisense oligonucleotides in db/db mice normalized plasma glucose levels [19]. Thus, a crucial role for PTEN in insulin sensitivity has been established.

Although silymarin is known to upregulate PTEN, causing its anti-tumor action, the effect of silymarin on insulin sensitivity remains unknown. In the present study, we used Wistar rats to evaluate the effect of silymarin on insulin sensitivity and clarify the role of PTEN in silymarin-induced insulin resistance.

## Materials and Methods

### Animals

Male Wistar rats weighing 200–250 g were purchased from the Animal Center of National Cheng Kung University Medical College. The rats were housed in a temperature-controlled room (25°C) and kept on a 12∶12 light/dark cycle (lights on at 6∶00 a.m.). Fructose-rich chow (Teklad Laboratory Diets, Madison, WI, USA) containing 60% fructose was fed for 4 weeks to induce insulin resistance according to our previously described method [20]. Development of insulin resistance in rats was characterized by loss of the tolbutamide-induced glucose-lowering lowering action. In brief, tolbutamide (10 mg/kg, i.p.) was injected into rats receiving fructose-rich chow to determine the change in blood glucose levels. Rats were treated independently through intragastric administration of silymarin (Sigma-Aldrich, St. Louis, MO, USA) dissolved in saline at 200 mg/kg once daily. Each rat was fasted overnight prior to all experiments. All animal procedures were performed according to the Guide for the Care and Use of Laboratory Animals of the National Institutes of Health and the guidelines of the Animal Welfare Act. The animal experiments were approved by the Regional Ethics Committee for Animal Research in Chi-Mei Medical Center (Tainan, Taiwan).

### Measuring the Body Weight and Biochemical Analysis

Body weights of the rats were measured first. Blood samples were collected from the femoral veins of rats under sodium pentobarbital anesthesia (30 mg/kg, i.p.), and serum was analyzed to determine levels of triglycerides, total cholesterol, low-density lipoprotein-cholesterol and high-density lipoprotein cholesterol using an automatic analyzer.

### Identification of Insulin Resistance

Insulin resistance was assessed using the homeostasis model assessment of insulin resistance (HOMA-IR) index according to previous reports [21], [22]. In brief, the HOMA-IR was calculated as fasting glucose (mmol/L)×fasting insulin (mU/L)/22.5. Plasma glucose concentrations were obtained using a commercial kit (Biosystems, Costa Brava, Barcelona, Spain) in an automatic analyzer (Quik-Lab; Ames, Miles Inc., Elkhart, IN, USA). Plasma insulin levels (pmol/L) were determined by an insulin enzyme-linked immunoassay (ELISA) kit (Mercodia AB, Uppsala, Sweden).

### Hyperinsulinemic-euglycemic Clamping

The hyperinsulinemic-euglycemic clamping was performed as described previously [23] with some modifications. Before the hyperinsulinemic-euglycemic clamping was perfomed, food was withdrawn for 12 h. Rats were then anesthetized with sodium pentobarbital (30 mg/kg, i.p.) and cannulated in the femoral vein for infusion of glucose and insulin and in the femoral artery for sampling. Animals were weighed and placed in a restrainer to which they were accustomed before the clamp procedure. At 0 min, rats were randomly assigned to be infused with regular human insulin (Novo Industries, Bagsvaerd, Denmark) at 40 mU/(kg·min). The infusate of insulin was diluted with saline containing 0.5% human serum albumin (Baxter, Glendale, CA, USA). Blood samples (10 µL) were withdrawn at 10-min intervals for the immediate determination of plasma glucose. On the basis of these values, 20% dextrose (Abbott, Chicago, IL, USA) was variably infused to maintain the plasma glucose concentration at approximately 5.5 mmol/L. The steady state was generally achieved within 70–90 min, whereupon a blood sample was collected to determine the glucose concentration. After terminal blood sampling at 120 min, the animals were killed with a lethal dose of sodium pentobarbital (100 mg/kg, i.p.). The glucose infusion rate at steady state was calculated using Steele’s equation [24].

### Cell Cultures

The L6 cell line was purchased from the American Type Culture Collection (Manassas, VA, USA). The cells were maintained at 37°C and 5% CO2 in Dulbecco’s modified Eagle’s medium (DMEM; HyClone, South Logan, UT, USA) supplemented with 10% fetal bovine serum (FBS). Cells (1×106) were plated on 60-mm culture dishes, and at 80% confluence, they were differentiated by culturing for 6–7 days in DMEM containing 2% fetal bovine serum. Medium was changed every other day.

### Glucose Uptake

A glucose uptake assay using 2-[*N*-(7-nitrobenz-2-oxa-1,3-diazol-4-yl)amino]-2-deoxy-d-glucose (2-NBDG; Invitrogen, Carlsbad, CA, USA) was performed according to a previously described method [25] with some modifications. Briefly, differentiated L6 cells were treated with or without a given concentration of silymarin and 1 µM insulin in the absence or presence of 200 µM 2-NBDG for 1 h. After the cells were washed, fluorescence intensity of 2-NBDG at 520–560-nm wavelength (480-nm excitation wavelength) was measured by the SPECTRA max 340PC ELISA reader (Molecular Devices Corporation, Union City, CA, USA). All cell culture results represent at least 2 independent experiments.

### Western Blotting Analysis

Total protein lysates from tissues or cells were extracted in lysis buffer (1% Triton X-100, 150 mM NaCl, 10 mM Tris [pH 7.5], and 5 mM ethylenediaminetetraacetic acid) containing a protease and phosphatase inhibitor cocktail (Sigma-Aldrich). Protein concentration was determined by the BCA assay kit (Pierce Biotechnology, Rockford, IL, USA). Protein lysates (50 µg) were separated using 10% sodium dodecyl-polyacrylamide gel electrophoresis and transferred to a polyvinylidene difluoride membrane (Millipore, Billerica, MA, USA). The membrane was blocked at 25°C for 1 h in Tris buffered saline with Tween (TBS-T; 10 mM Tris [pH 7.6], 150 mM NaCl, and 0.05% Tween 20), containing 3% bovine serum albumin, and probed with primary antibodies (1∶1000), such as those against PTEN, phospho-Akt, and Akt (Cell Signaling Technology, Beverly, MA, USA) at 4°C overnight. After the membrane had been washed with TBS-T, the blots were incubated with a 1/5000 dilution of horseradish peroxidase-conjugated secondary antibodies at 25°C for 1 h. Protein bands were visualized using the enhanced chemiluminescence kit (PerkinElmer, Boston, MA, USA). Actin (Millipore) was used as an internal control. The optical densities of the bands were determined using software (Gel-Pro Analyzer version 4.0 software (; Media Cybernetics Inc., Silver Spring, MD, USA).

### Small Interfering Ribonucleic Acid Transfection

L6 cells were grown in 6-well culture plates at a density of 2×105 cells/well and transfected with duplexed RNA oligonucleotides (Stealth RNAi™; Invitrogen) of PTEN or scrambled small interfering RNA (siRNA) (as negative control) using Lipofectamine 2000TM (Invitrogen) according to the manufacturer’s instructions. The cells were used at 48-h after-transfection.

### Statistical Analysis

Data are expressed as means ± standard error of means. An analysis of variance and Dunnett’s post hoc range comparisons were used to determine the source of significant differences where appropriate. Significance is defined as a P-value <0.05.

## Results

### Silymarin Induces Insulin Resistance in Wistar Rats

Oral administration of silymarin at 200 mg/kg body weight once daily for 14 days as described previously [25] produced insulin resistance in the Wistar rats, which was identified using the HOMA-IR or hyperinsulinemic-euglycemic clamping. In preliminary experiments, various doses of silymarin were administered to Wistar rats, and we found that 200 mg/kg body weight as most reliable dose to induce insulin resistance in rats. Therefore, we used this dose in all following studies. No intolerance, side effects, or allergic reactions were observed.

**Figure 1:**
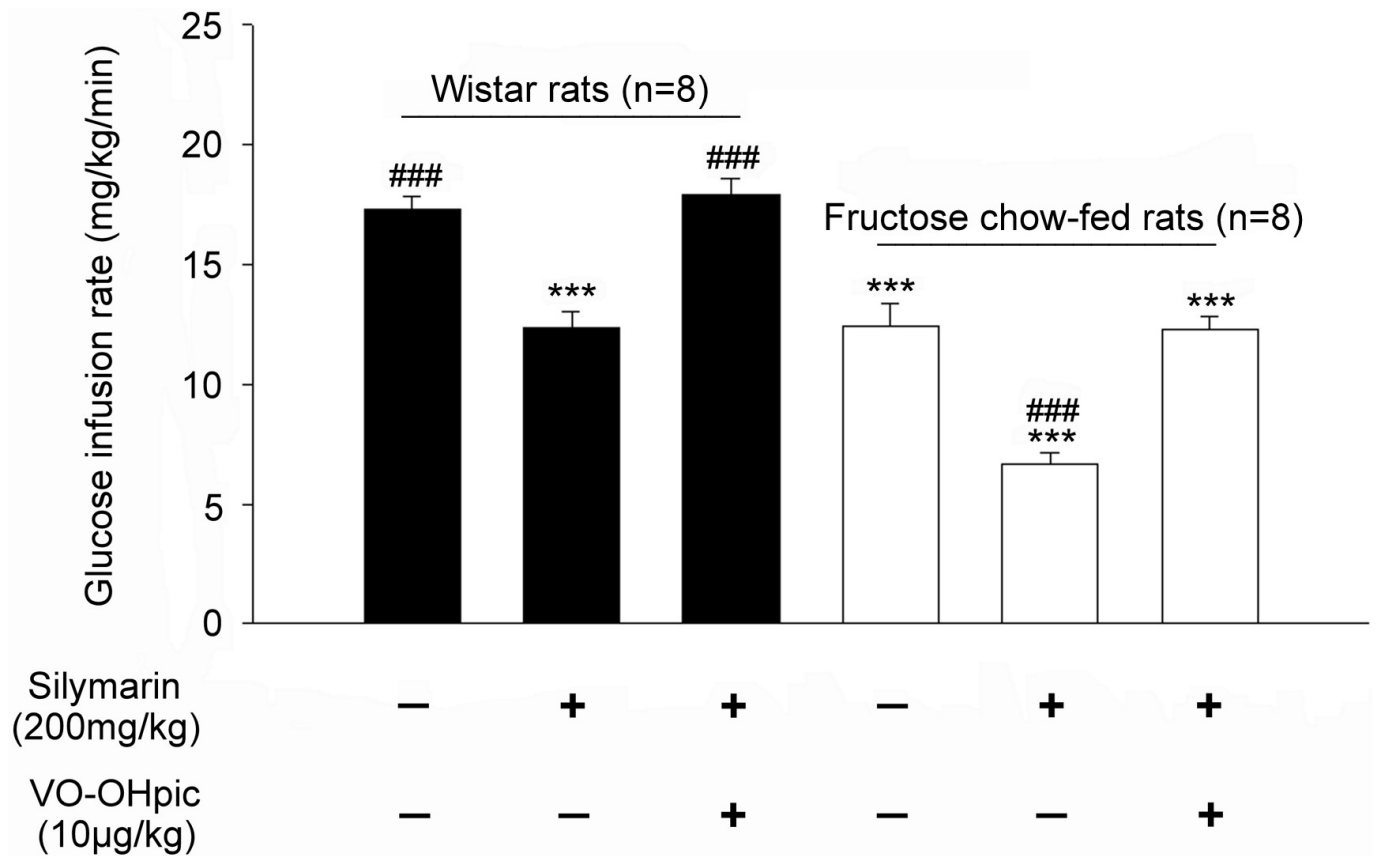
Mean values for the direct measure of insulin sensitivity, as assessed by the glucose infusion rate during the last 20-h hyperinsulinemic-euglycemic clamp. Silymarin (200 mg/kg body weight) was orally administered once daily to Wistar rats for 2 weeks. A glucose clamp was performed 30 min after the oral intake of silymarin (200 mg/kg) in Wistar rats fed normal or fructose-rich chow. VO-OHpic (PTEN inhibitor, 10 µg/kg) was i.p. injected 30 min before administration of silymarin. The vehicle used to dissolve the testing drugs was given at the same volume. Values (mean ± SE) were obtained from each group of 8 animals. ***P<0.001 compared with vehicle-treated normal chow-fed group. ^###^ *P*<0.001 compared with the vehicle-treated fructose-rich chow-fed group.

In rats receiving fructose-rich chow for the induction of insulin resistance, similar treatment with silymarin significantly enhanced insulin resistance (Tab. 1). In addition to HOMA-IR, the exacerbation of insulin resistance by silymarin in rats receiving fructose-rich chow was also evident by the hyperinsulinemic-euglycemic clamping method (Fig. 1).

**Table 1:** Effects of silymarin on body weight, biochemical indicators and HOMA-IR) in Wistar rats receiving fed fructose-rich chow or normal chow.

	HOMA-IRindex	Cholesterol(mg/dL)	TG(mg/dL)	LDL(mg/dL)	HDL(mg/dL)	LDL/HDL	Body weight
**Normal chow-fed rats (n = 8)**							
** +Vehicle**	6.47±0.27	97.13±7.22	93.13±15.17	63.37±6.44	16.88±2.11	4.12±0.79	288.20±6.27
** +Silymarin (200 mg/kg)**	13.00±0.51	100.38±10.52	116.38±32.62	61.10±11.62	16.00±2.01	4.27±1.05	288.42±8.45
**Fructose-rich chow-fed rats (n = 8)**							
** +Vehicle**	18.68±0.49***	111.45±8.78	176.25±22.79**	63.83±5.95	12.38±2.46	6.35±1.06	304.62±10.96
** +Silymarin (200 mg/kg)**	21.82±0.62*** ^##^	116.43±11.35	175.38±23.66**	62.55±9.83	12.50±2.76	6.36±1.49	305.91±12.12

Silymarin (200 mg/kg body weight) was orally administerated once daily to Wistar rats fed fructose-rich or normal chow for 2 weeks. The HOMA-IR index was calculated as fasting glucose (mmol/L)×fasting insulin (mU/L)/22.5. Data represent mean ± standard error of mean of 8 animals.

*P*<0.001 compared with normal chow-fed rats receiving vehicle;

^###^ *P*<0.001 compared with fructose-rich chow-fed rats receiving vehicle.

Interestingly, this action of silymarin was impeded by a PTEN inhibitor (VO-OHpic) at the effective dose as described in a previous report [26]. Moreover, this action of silymarin disappeared within 2 or 3 days following the termination of dosing in rats. Thus, an irreversible action of silymarin was negligible.

### Increased PTEN Expression by Silymarin in Wistar Rats

**Figure 2:**
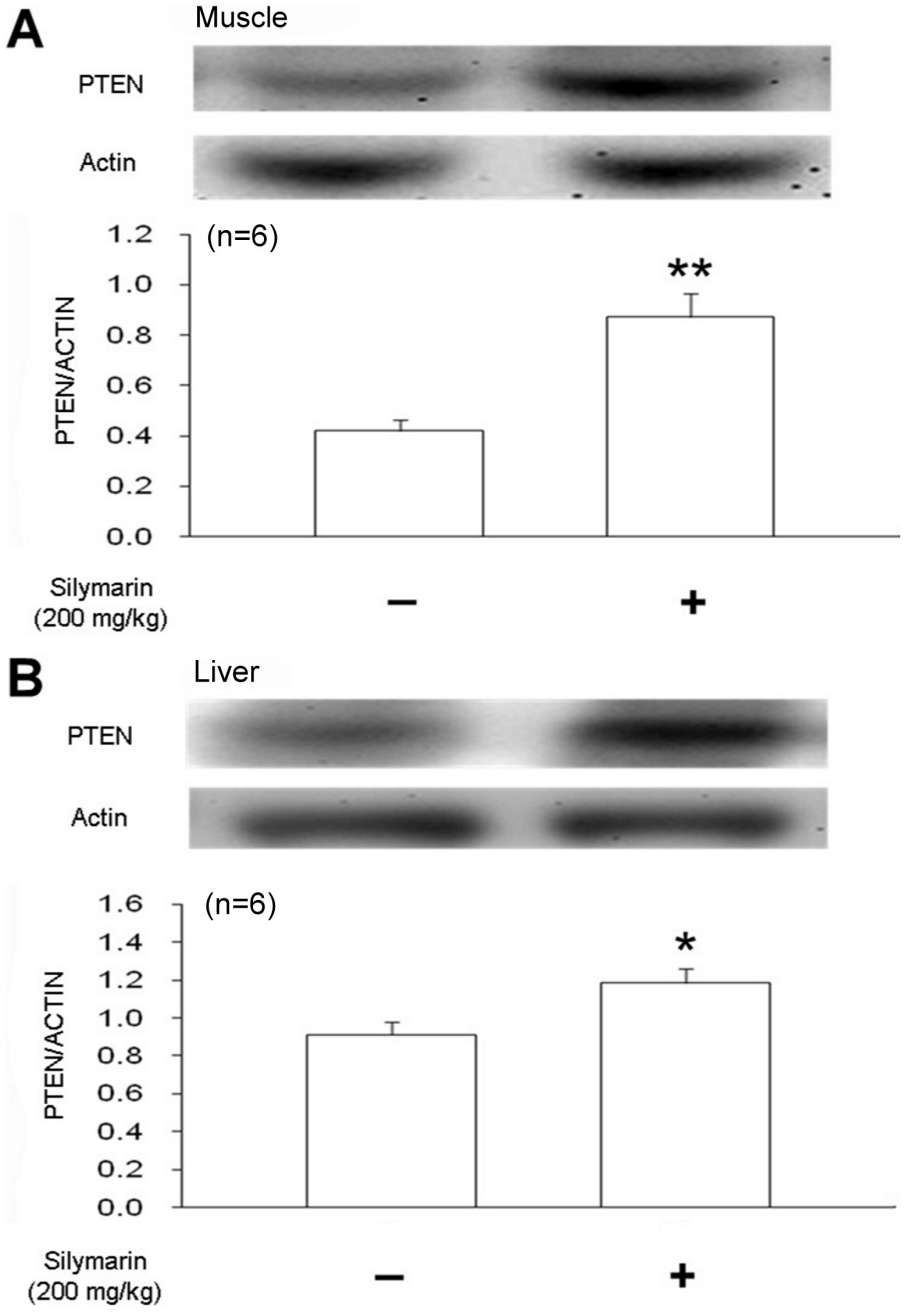
The effect of silymarin on the expression of PTEN. Silymarin (200 mg/kg body weight) was orally administered once daily to Wistar rats for 2 weeks. Skeletal muscle (A) and liver (B) were isolated for identification of PTEN expression using western blotting analysis. Data are obtained from 6 individual experiments and expressed as mean ± standard error of mean. *P<0.05; **P<0.01 compared with the vehicle-treated group (first column).

Skeletal muscle and the liver are widely used to identify insulin resistance. Therefore, both tissues were used to investigate the changes in PTEN expression using western blotting analysis. Silymarin significantly increased PTEN expression in skeletal muscle and the liver as shown in Fig. 2; PTEN expression was increased 2.03-fold by silymarin in skeletal muscle and 1.27-fold the in the liver.

### Silymarin Disrupts Insulin Signaling to Decrease Glucose Uptake in Cultured L6 Myotube Cells

**Figure 3:**
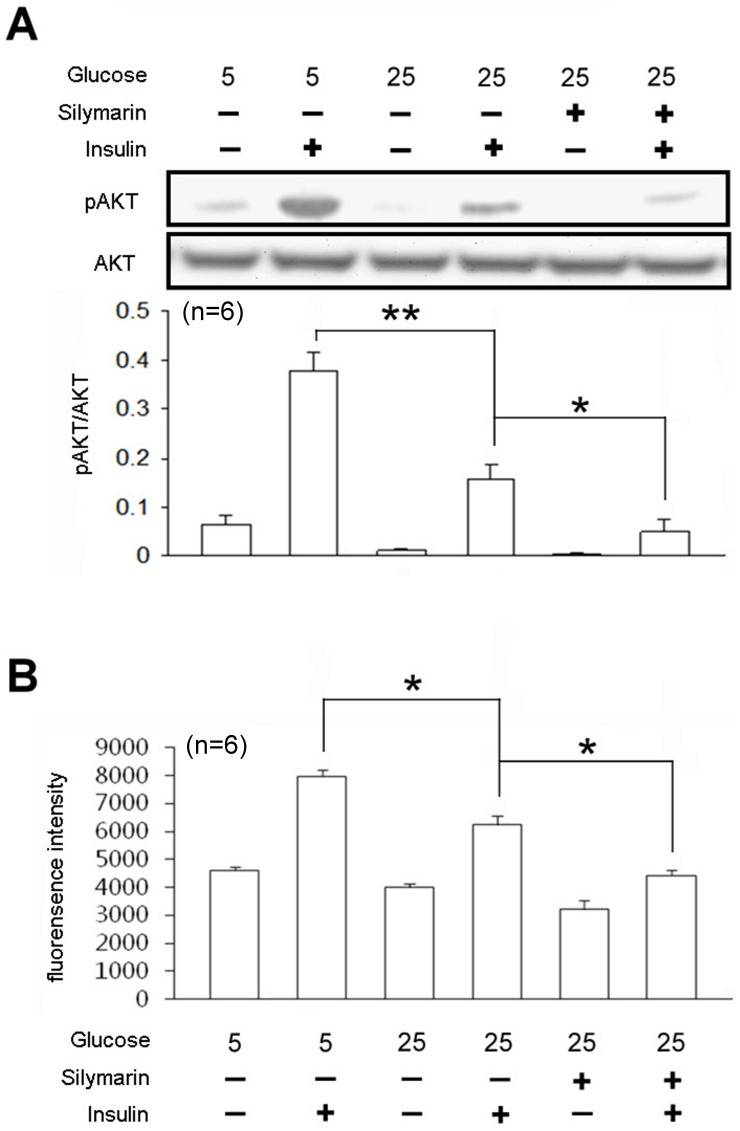
Silymarin disrupted insulin signaling to decrease glucose uptake in L6 myotube cells. L6 cells were maintained in normal-glucose (5 mM) or high-glucose (25 mM) medium. Cells were treated with 1 µM silymarin, and each group was exposed or not to 0.1 µM insulin for 30 min. Equal amounts of the total lysates of each group were immunoblotted with anti-phospho-Akt (pAkt) or Akt (A). The L6 cells were then treated with 200 µM 2-NBDG for 10 min to evaluate the glucose uptake ability of each group (B). Values represent means ± standard error of mean of 3 independent experiments. *P<0.05; **P<0.01 compared with the control group.

Insulin significantly increased the phosphorylation of Akt in cultured L6 cells, maintained in low-glucose condition (5 mM), whereas the elevated phosphor-Akt levels were decreased in high-glucose (25 mM) condition, implying the development of insulin resistance in cells. Pretreatment with silymarin aggravated high glucose-induced insulin resistance (Fig. 3A). To further confirm the development of insulin resistance, glucose uptake ability was assessed. Decreased glucose uptake was observed in cells maintained in high-glucose conditions and silymarin exacerbated this effect of high glucose on glucose uptake (Fig. 3B).

### Deletion of PTEN in Cultured L6 Myotube Cells Reversed Silymarin-induced Insulin Resistance

**Figure 4:**
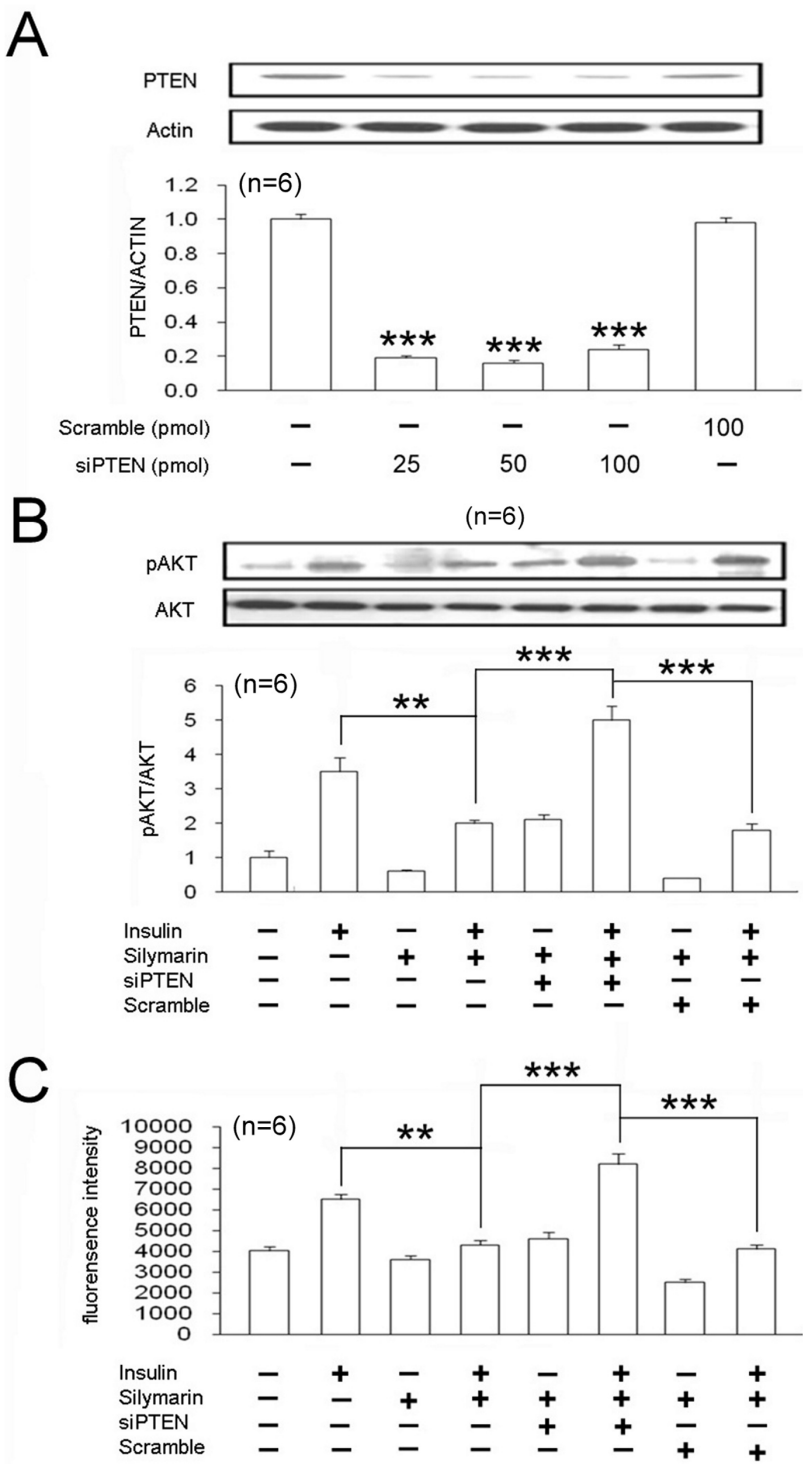
PTEN deletion in L6 cells reversed the effect of silymarin on insulin sensitivity and glucose uptake. Cells were transfected for 48(A). Cells treated with 1 µM silymarin for 6 h with or without specific PTEN siRNA were exposed or not to 0.1 µM insulin. Equal amounts of the total lysates of each group were immunoblotted with anti-phospho-Akt (pAkt) or Akt (B). Cells were transfected for 48 h with 50 pmol PTEN siRNA or scramble siRNA were treated with 1 µM silymarin 6 h with or without specific PTEN siRNA and exposed or not to 0.1 µM insulin for 30 min. The L6 cells were then treated with 200 µM 2-NBDG for 1 h to evaluate the glucose uptake ability of each group (C). Values represent means ± standard error of mean for 3 independent.

To confirm the role of PTEN in silymarin-induced insulin resistance, siRNA specific to PTEN was used. Transfection with this specific siRNA for PTEN significantly decreased the expression of PTEN in L6 cells, whereas scrambled siRNA had no effect (Fig. 4A). Disruption of insulin signaling by silymarin was reversed in L6 cells with specific PTEN deletion, whereas scrambled siRNA failed to affect the silymarin-induced effects (Fig. 4B).

Moreover, glucose uptake showed similar changes. In the presence of PTEN-specific siRNA, the effect of silymarin on glucose uptake was significantly reversed in L6 cells, wheras scrambled siRNA was not effective (Fig. 4C).

## Discussion

In the present study, we found that silymarin induced insulin resistance in normal rats and enhanced insulin resistance in rats receiving fructose-rich chow. Insulin resistance was characterized using the HOMA-IR and hyperinsulinemic-euglycemic clamping. HOMA-IR is widely used clinically to identify the presence of insulin resistance [27]–[29], and hyperinsulinemic-euglycemic clamping is reported as a reliable tool in research for the estimation of insulin resistance in animals [30], [31]. To the best of our knowledge, this is the first study to show that silymarin may induce insulin resistance. In this report, we demonstrated the presence of insulin resistance using HOMA-IR as described previously [32] while the values were different with that in clinics [28], [29]. In addition, the effective dose in rats was not the same as that used in clinics; oral dose of silymarin in human subjects was 420–1680 mg/day [33], [34] whereas the dose for cancer-resistance was 13000 mg/day [35].

It has been established that silymarin is a safe and well-tolerated dietary food supplement [4] and is widely used to protect against liver damage induced by chemicals [36], [37]. In addition, the ability of silymarin to lower blood glucose and cholesterol has been documented in type-1 like diabetic rats [38]. However, another study indicated that silymarin had no significant effect on blood glucose [39]. Therefore, the effect on silymarin on blood glucose in diabetic animals remains obscure. The difference seems related to the dose used in the animals as the anti-diabetic action of silymarin was observed in lower doses through antioxidant-like actions [38]. Indeed, silymarin shows multiple actions related to the treatment dose [40], [41]. Thus, silymarin-induced insulin resistance seems different from that described in previous reports [42], [43] owing to the variation in dosing.

The anti-cancer action of silymarin is associate with an increase of PTEN [13]. Moreover, PTEN specific deletion in muscle improves skeletal muscle insulin sensitivity and protects mice from insulin resistance [18]. A crucial role for PTEN in insulin sensitivity has been documented [44], [45]. Interesting, the effect of silymarin was reduced by a PTEN inhibitor, VO-OHpic, which has been used in previous studies [26], [46]. Furthermore, PTEN expression identified by western blot was markedly increased by silymarin (Fig. 2), Thus revealing mediation of PTEN in the action of silymarin.

Glucose uptake in skeletal muscle plays an important role in glucose homeostasis [47], and we used L6 cells to investigate the potential underlying mechanism(s). Because isolated skeletal muscle preparations are short-lived during experimental manipulation [48] and nedds to be obtained from freshly killed animals, we used L6 cells as an alternative to investigate glucose uptake. Radioactive tracers are also widely employed glucose uptake assays [49]. To avoid radioactive damage, a fluorescence indicator, 2-NBDG, has been developed [50], and is generally measured by a fluorescence spectrofluorometer [51] or by an ELISA reader [52]. Similar to previous studies [25], [53], we determined the changes in 2-NBDG uptake using an ELISA reader.

High glucose significantly impaired insulin signaling in L6 cells, whereas silymarin augmented high glucose-induced insulin resistance (Fig. 3A). To confirm the development of insulin resistance, glucose uptake in L6 cells was investigated. In parallel with the impaired insulin signaling as shown in Fig. 3A, glucose uptake was also impaired in silymarin-treated L6 cells under the high glucose condition, similar to that observed in rats receiving fructose-rich chow. It has been reported that silymarin induces the expression/activity of PTEN, [13] and that PTEN is responsible for the disruption of insulin signaling [15]. This view is consistent with our data that deletion of PTEN using siRNA reverses both silymarin-induced insulin resistance and impaired glucose uptake in L6 cells (Fig. 4). There is no doubt that the action of silymarin is produced though a PTEN-dependent pathway. However, the present study is mainly focused on the insulin sensitivity in muscle. Effect of silymarin on the insulin signals in liver shall be characterized in the future.

Taken together, PTEN loss is considered as a biomarker for activated PI3K/AKT, a pathway frequently mutated in cancer [16]. A previous study has demonstrated that silymarin could inhibit the Akt signaling pathway through increased PTEN expression, indicating its potential for suppressing migration and invasion of cancer cells [54]. Therefore, we should be aware of the possibility of insulin resistance when using silymarin for cancer therapy in patients with diabetes or with family history of diabetes.

In conclusion, we suggest that silymarin induces insulin resistance in Wistar rats and enhances insulin resistance in rats fed fructose-rich chow through an increase of PTEN. Administration of silymarin to cancer patients may affect insulin sensitivity, which should not be ignored clinically.
